# Modelling carbon dynamics from urban land conversion: fundamental model of city in relation to a local carbon cycle

**DOI:** 10.1186/1750-0680-1-8

**Published:** 2006-08-15

**Authors:** Anastasia Svirejeva-Hopkins, Hans-Joachim Schellnhuber

**Affiliations:** 1 University of Lisbon, Faculty of Sciences, IDL, Observatório Astronómico de Lisboa Edifício Leste, Tapada da Ajuda 1349-018 Lisbon, Portugal; 2 Potsdam Institute for Climate Impact Research, P.O.Box 6012, Telegrafenberg D-14412, Germany

## Abstract

**Background:**

The main task is to estimate the qualitative and quantitative contribution of urban territories and precisely of the process of urbanization to the Global Carbon Cycle (GCC). Note that, on the contrary to many investigations that have considered direct anthropogenic emission of CO_2_(urbanized territories produce ca. 96–98% of it), we are interested in more subtle, and up until the present time, weaker processes associated with the conversion of the surrounding natural ecosystems and landscapes into urban lands. Such conversion inevitably takes place when cities are sprawling and additional "natural" lands are becoming "urbanized".

**Results:**

In order to fulfil this task, we first develop a fundamental model of urban space, since the type of land cover within a city makes a difference for a local carbon cycle. Hence, a city is sub-divided by built-up, „green" (parks, etc.) and informal settlements (*favelas*) fractions. Another aspect is a sub-division of the additional two regions, which makes the total number reaching eight regions, while the UN divides the world by six. Next, the basic model of the local carbon cycle for urbanized territories is built. We consider two processes: carbon emissions as a result of conversion of natural lands caused by urbanization; and the transformation of carbon flows by "urbanized" ecosystems; when carbon, accumulated by urban vegetation, is exported to the neighbouring territories. The total carbon flow in the model depends, in general, on two groups of parameters. The first includes the NPP, and the sum of living biomass and dead organic matter of ecosystems involved in the process of urbanization, and namely them we calculate here, using a new more realistic approach and taking into account the difference in regional cities' evolution.

**Conclusion:**

There is also another group of parameters, dealing with the areas of urban territories, and their annual increments. A method of dynamic forecasting of these parameters, based on the statistical regression model, was already suggested; nevertheless we shall further develop a new technique based on one idea to use the gamma-distribution. This will allow us to calculate the total carbon balance and to show how urbanization shifts it.

## Background

This article represents one of the consecutive publications dedicated to the following problem: how much does the urbanisation process influence the Global Carbon Cycle (GCC), which was begun by the work [1]. At present there are a lot of different models, which describe various aspects of the GCC, as well as estimates of the values of anthropogenic carbon emissions and terrestrial uptake [2,3]. Since the GCC's functioning involves the great variety of feedback mechanisms and responses, there is a growing need to focus not only on the simple emissions-atmosphere relationship, but also to take into consideration the other aspects of the carbon cycle, subtle local processes, feedbacks and non-linearities [4].

Note that compared to most of the research concerning anthropogenic CO_2 _emissions, in this paper we do not consider the direct emissions, while it is known that urbanised territories produce about 96–98% of them [5]. We would rather focus on subtler and, up until the present time, weaker processes, caused by the land conversion of natural ecosystems and landscapes. Such conversion inevitably takes place when cities are sprawling, with additional "natural" lands becoming "urbanised". Certainly, because of their relatively small size (some authors have estimated the total area of urbanised territories in the 1980s as occupying only 1–2% of the total land area [6,7], their role could also be presumed small and their impact on the GCC negligible. One particular argument is that the area of urbanised territories is relatively insignificant when compared to the total territory participating in the GCC. Nevertheless, the growth of world population, global energy consumption and the growth of urban populations are characterized by exponential and even hyperbolic growth (especially for a certain finite time periods), when the factor being negligibly small at present could become significantly important in the near future. Therefore, we should consider the dynamics of urbanisation in order to assess its influence on the GCC.

In relation to the local carbon cycle, the total city's area cannot be considered as homogenous. For example, while a "built up" area is disconnected from the carbon cycle, another, so-called "green" area (open space, covered by parks and recreation territories) [8] continues to participate in the processes of carbon accumulation and exchange (although, probably, with altered characteristics). Apparently, it is possible to describe the alteration of land-use on urban territory by the change of the relationship between these two areas. Thus, the expression "urbanised territory" does not automatically imply that the entire green surface of a natural territory of surrounding biomes is transformed into one, covered totally by buildings, roads etc; some part remains "green" and continues to function as an ecosystem. Its characteristics and types of functioning, however, become very different, i.e. it is now an "urbanised ecosystem". In particular, not only the quantities but also the qualities of the carbon fluxes change significantly in this ecosystem.

Naturally, the quantitative estimation of the "green" area depends to a large extent on the type of urbanisation, which has occurred, for example, the plan (or lack of) for city growth, regulations and laws, the attractiveness of a city for a rural population and "*favelisation*", i.e. the growth of informal settlements [9]. As to the functioning of urban territory in the local carbon cycle then, in general, we may consider it as a surface with specific rates of CO_2 _– uptake and emission, which in turn depend on city's structure. In other words, we "spread" the different types of city's surfaces over the whole city area, in a way that any area unit possesses all properties of these surfaces ("built up", "green", "*favelas*").

As a result, we shall develop a spatial model of the organisation of city's area: "fundamental city model", and calculate with the help of it the regional values of city sub-areas, mean regional values of NPP; biomass and the dead organic matter for urbanised territories of the world.

## Results

### Structure of the city's area

Naturally, buildings, roads, concrete and asphalt do not cover the whole urban surface; there are also comparatively large segments of land covered by trees, shrubs and grass in the form of parks, gardens, lawns, etc. All of these are called the "green city area" or the "free city space". The green area is a mosaic of many quasi-natural micro-ecosystems and plays the main role in the biological part of the local carbon cycle of the ecosystem "city".

In the 1970s, the concept of "city open space plan" was introduced [8]. In accordance with this concept, the whole city area is divided into two fractions *p*_1 _and *p*_2 _(*p*_1 _+ *p*_2 _= 1): (1) developed or "built-up" area (residential, commercial, industrial, institutional, and roads), i.e. an area covered by artificial surfaces without vegetation; (2) undeveloped area or "free space" (waste disposal and recreational parks) partially covered by vegetation. It is natural that a quantitative relation between these fractions is different for different regions of the world. We use the standard UN regional subdivision [9], modified in [1], (see table 1). E. Odum showed that for a typical American city, the free space was 71% in the 1970s. If no urban planning is applied, then the free space will be reduced to 16% by the year 2000. Judicious planning of residential and other development can preserve a third of the area as free space, including adequate space for efficient semi-natural tertiary treatment of both industrial and domestic wastes in ponds and well-planned landfills located in the larger waste disposal parks.

**Table 1:** Regional subdivision of the world and their denominations

1. Africa (excluding North) – **Afr**
2. Arabian Countries – **Ar**
3. China – Cn
4. Asia and Pacific (excluding Near and Middle East) – **AsP**
5. Latin America and Caribbean – **LAC**
6. Countries with Economy in Transition (FSU and East Europe) – **ET**
7. Highly Industrialised Countries in Europe – **HI**
8. USA, Canada, Australia and New Zeeland – **UCA**

Hence, we assume that the relative "green" area, *p*_*g*_, for the UCA region is 33% (*p*_*g *_= 0.33). For Germany on the other hand, free space in a city is estimated to be 18.2%. This is a mean value calculated for 116 German cities [10,11], but this does not take into account the vegetation of residence quarters. For the European cities overall, 5% of the residence quarters' area is covered by woodland, and 25% by grass [12]. Since residence quarters comprise 38% of a total city space, we obtain a value of (18.2 + 38·(0.05 + 25))%≈ 30% (*p*_*g *_= 0.3) for the green area of German cities in the 1990s. We will assume that this value may be taken as a relative estimate of city green areas for the HI and UCA regions (Tab 1).

We also assume that the value of *p*_1 _must be the same for the cities of all eight regions. The following concept justifies this assumption. Any city is a complex social system, and its spatial structure is adjusted for the normal functioning of a city. Therefore, integral structural characteristics such as the relative area occupied by various subsystems that provide the normal functioning of a city (industry, service, municipal institutions, roads, etc.) must be general system invariant. It does neither depend (or, only weakly depends) upon the economic status of the region, nor upon its specific cultural characteristics. Also, since we have already supposed that any city grows similarly, then the invariant does not depend on time either.

Therefore, while the relative part of the "built-up" area remains constant, the "free space" area can be redistributed between the "green" and the area occupied by so-called "informal" low-income settlements, abundant in the developing world. Nowadays, informal settlements are the ordinary phenomena of urbanisation in many regions of the World. These settlements, like inner-city slums, are called *favelas *or *tugurios *in Latin America, *chawls *in India and shop-house tenements in South-East Asia. From now on, we shall use the common word *favelas*. Regarding their role in the carbon cycle, on the one hand, *favelas *do not have any green plants on their territory, while on the other, they tend to produce lower emissions, and have a more compact structure than conventional built-up areas.

We presuppose that the territory of informal settlements can expand only at the expense of the green territories. This is explained by the fact that parks and other urban recreation areas usually belong to municipalities, where the property rights appear to be not as strict compared with private ownership.

The free city space area, *p*_2_, be represented as:

*p*_2 _= *p*_*f *_+ *p*_*g *_= *fp*_2 _+ (1 - *f*)*p*_2 _    (1)

where *p*_*f *_= *fp*_2 _is the fraction of city area occupied by informal settlements and *p*_*g *_= (1 - *f*)*p*_2 _is the fraction of green (covered by vegetation) area. As it was mentioned earlier, *p*_2 _= 0.3, while *f *is the coefficient of "*favelisation*" (0 <*f *< 1). Evidently, for the HI and UCA regions,*f *= 0.

It is quite a difficult task to collect reliable statistics on *favelas' *areas. The existing sources were very scarce (for instance, [9]), therefore, we had to make several additional assumptions. As a result, the following average estimations of the percentages of *favelas' *areas and *f *(coefficient of *favelisation*) are presented in Tab. 2. At the moment, we cannot provide a detailed prognosis of *favelas' *dynamics, therefore the hypothesis is that the values of *f *are constant.

**Table 2:** Relative green, *p*_*g *_, and favelas, *p*_*f *_, areas within a city

Region	Afr	***Ar***	***Asp***	***Cn***	***LAC***	***ET***	***HI***	***UCA***
*p*_*f*_%	15	10	12	1	20	1	0	0
*p*_*g*_%	15	20	18	29	10	29	30	30
*f*	0.50	0.33	0.40	0.03	0.67	0.03	0.0	0.0

Finally, for each region *j*(*j *= 1,2,...8) the area of an urbanised territory *S*^*j *^can be presented as a sum of three items:

*S*^*j *^= Sbj
 MathType@MTEF@5@5@+=feaafiart1ev1aaatCvAUfKttLearuWrP9MDH5MBPbIqV92AaeXatLxBI9gBaebbnrfifHhDYfgasaacH8akY=wiFfYdH8Gipec8Eeeu0xXdbba9frFj0=OqFfea0dXdd9vqai=hGuQ8kuc9pgc9s8qqaq=dirpe0xb9q8qiLsFr0=vr0=vr0dc8meaabaqaciaacaGaaeqabaqabeGadaaakeaacqWGtbWudaqhaaWcbaGaemOyaigabaGaemOAaOgaaaaa@30B2@ + Sgj
 MathType@MTEF@5@5@+=feaafiart1ev1aaatCvAUfKttLearuWrP9MDH5MBPbIqV92AaeXatLxBI9gBaebbnrfifHhDYfgasaacH8akY=wiFfYdH8Gipec8Eeeu0xXdbba9frFj0=OqFfea0dXdd9vqai=hGuQ8kuc9pgc9s8qqaq=dirpe0xb9q8qiLsFr0=vr0=vr0dc8meaabaqaciaacaGaaeqabaqabeGadaaakeaacqWGtbWudaqhaaWcbaGaem4zaCgabaGaemOAaOgaaaaa@30BC@ + Sfj
 MathType@MTEF@5@5@+=feaafiart1ev1aaatCvAUfKttLearuWrP9MDH5MBPbIqV92AaeXatLxBI9gBaebbnrfifHhDYfgasaacH8akY=wiFfYdH8Gipec8Eeeu0xXdbba9frFj0=OqFfea0dXdd9vqai=hGuQ8kuc9pgc9s8qqaq=dirpe0xb9q8qiLsFr0=vr0=vr0dc8meaabaqaciaacaGaaeqabaqabeGadaaakeaacqWGtbWudaqhaaWcbaGaemOzaygabaGaemOAaOgaaaaa@30BA@,     (2)

where Sbj
 MathType@MTEF@5@5@+=feaafiart1ev1aaatCvAUfKttLearuWrP9MDH5MBPbIqV92AaeXatLxBI9gBaebbnrfifHhDYfgasaacH8akY=wiFfYdH8Gipec8Eeeu0xXdbba9frFj0=OqFfea0dXdd9vqai=hGuQ8kuc9pgc9s8qqaq=dirpe0xb9q8qiLsFr0=vr0=vr0dc8meaabaqaciaacaGaaeqabaqabeGadaaakeaacqWGtbWudaqhaaWcbaGaemOyaigabaGaemOAaOgaaaaa@30B2@ = (1 - *p*_2_)*S*^*j *^= 0.7*S*^*j *^is the built-up area, Sgj
 MathType@MTEF@5@5@+=feaafiart1ev1aaatCvAUfKttLearuWrP9MDH5MBPbIqV92AaeXatLxBI9gBaebbnrfifHhDYfgasaacH8akY=wiFfYdH8Gipec8Eeeu0xXdbba9frFj0=OqFfea0dXdd9vqai=hGuQ8kuc9pgc9s8qqaq=dirpe0xb9q8qiLsFr0=vr0=vr0dc8meaabaqaciaacaGaaeqabaqabeGadaaakeaacqWGtbWudaqhaaWcbaGaem4zaCgabaGaemOAaOgaaaaa@30BC@ = *p*_2_(1 - *f*^*j*^)*S*^*j *^= 0.3(1 - *f*^*j*^)*S*^*j *^is the green area and Sfj
 MathType@MTEF@5@5@+=feaafiart1ev1aaatCvAUfKttLearuWrP9MDH5MBPbIqV92AaeXatLxBI9gBaebbnrfifHhDYfgasaacH8akY=wiFfYdH8Gipec8Eeeu0xXdbba9frFj0=OqFfea0dXdd9vqai=hGuQ8kuc9pgc9s8qqaq=dirpe0xb9q8qiLsFr0=vr0=vr0dc8meaabaqaciaacaGaaeqabaqabeGadaaakeaacqWGtbWudaqhaaWcbaGaemOzaygabaGaemOAaOgaaaaa@30BA@ = *p*_2_*f*^*j*^*S*^*j *^= 0.3*f*^*j*^*S*^*j *^is the area occupied by *favelas*.

### Land use model

#### Losses of organic carbon

Let the area of urbanised territory of the *j*^*th*^region in the year *t *be equal to *S*^*j*^(*t*). Its annual increments, which may be interpreted as an annual rate of city's "sprawling", equals to Δ*S*^*j *^(*t*) = *S*^*j *^(*t*) - *S*^*j *^(*t *- 1). We assume that this area, occupied by surrounding natural ecosystems with local densities of living biomass, (*B*^*j*^), and dead organic matter (humus), (*D*^*j*^), is replaced by "built-up" and "green" areas in the proportion (1 - pgj
 MathType@MTEF@5@5@+=feaafiart1ev1aaatCvAUfKttLearuWrP9MDH5MBPbIqV92AaeXatLxBI9gBaebbnrfifHhDYfgasaacH8akY=wiFfYdH8Gipec8Eeeu0xXdbba9frFj0=OqFfea0dXdd9vqai=hGuQ8kuc9pgc9s8qqaq=dirpe0xb9q8qiLsFr0=vr0=vr0dc8meaabaqaciaacaGaaeqabaqabeGadaaakeaacqWGWbaCdaqhaaWcbaGaem4zaCgabaGaemOAaOgaaaaa@30F6@) : pgj
 MathType@MTEF@5@5@+=feaafiart1ev1aaatCvAUfKttLearuWrP9MDH5MBPbIqV92AaeXatLxBI9gBaebbnrfifHhDYfgasaacH8akY=wiFfYdH8Gipec8Eeeu0xXdbba9frFj0=OqFfea0dXdd9vqai=hGuQ8kuc9pgc9s8qqaq=dirpe0xb9q8qiLsFr0=vr0=vr0dc8meaabaqaciaacaGaaeqabaqabeGadaaakeaacqWGWbaCdaqhaaWcbaGaem4zaCgabaGaemOAaOgaaaaa@30F6@. The latter remains the same as before and is occupied by the natural ecosystems, while all living biomass in the first part is completely destroyed, relatively quickly decomposed, and emitted into the atmosphere in the form of CO_2_. Since any construction of roads and buildings is accompanied by the destruction of soil structure, its fragmentation and the increase in its aeration, it will likewise result in the destruction of soil humus, which will also be emitted into the atmosphere as CO_2_. This is a typical process of the *land conversion*. Thus, the annual amount of carbon emitted by the *j*^*th *^region (the annual carbon outflow) is equal to:

dClj(t)=(1−pgj)ΔSj(t)⋅⌊(Bj)*+(Dj)*⌋     (3)
 MathType@MTEF@5@5@+=feaafiart1ev1aaatCvAUfKttLearuWrP9MDH5MBPbIqV92AaeXatLxBI9gBaebbnrfifHhDYfgasaacH8akY=wiFfYdH8Gipec8Eeeu0xXdbba9frFj0=OqFfea0dXdd9vqai=hGuQ8kuc9pgc9s8qqaq=dirpe0xb9q8qiLsFr0=vr0=vr0dc8meaabaqaciaacaGaaeqabaqabeGadaaakeaacqWGKbazcqWGdbWqdaqhaaWcbaGaemiBaWgabaGaemOAaOgaaOGaeiikaGIaemiDaqNaeiykaKIaeyypa0JaeiikaGIaeGymaeJaeyOeI0IaemiCaa3aa0baaSqaaiabdEgaNbqaaiabdQgaQbaakiabcMcaPiabfs5aejabdofatnaaCaaaleqabaGaemOAaOgaaOGaeiikaGIaemiDaqNaeiykaKIaeyyXIC9aayWaceaacqGGOaakcqWGcbGqdaahaaWcbeqaaiabdQgaQbaakiabcMcaPmaaCaaaleqabaGaeiOkaOcaaOGaey4kaSIaeiikaGIaemiraq0aaWbaaSqabeaacqWGQbGAaaGccqGGPaqkdaahaaWcbeqaaiabcQcaQaaaaOGaayj84laawUp+aiaaxMaacaWLjaWaaeWaceaacqaIZaWmaiaawIcacaGLPaaaaaa@5C32@

where (1 - pgj
 MathType@MTEF@5@5@+=feaafiart1ev1aaatCvAUfKttLearuWrP9MDH5MBPbIqV92AaeXatLxBI9gBaebbnrfifHhDYfgasaacH8akY=wiFfYdH8Gipec8Eeeu0xXdbba9frFj0=OqFfea0dXdd9vqai=hGuQ8kuc9pgc9s8qqaq=dirpe0xb9q8qiLsFr0=vr0=vr0dc8meaabaqaciaacaGaaeqabaqabeGadaaakeaacqWGWbaCdaqhaaWcbaGaem4zaCgabaGaemOAaOgaaaaa@30F6@) = 0.7 + 0.3*f*^*j*^, and the values for *f*^*j *^are taken from Tab. 2.

#### Redistribution of carbon flows by "urbanized" ecosystem

If we look at a standard picture of the carbon flows in any territory we can see that these flows are divided into two groups: (a) vertical exchange flows between the atmosphere and the surface, and (b) horizontal exchange flows between the given territory and other neighboring areas. In natural terrestrial ecosystems, the horizontal flows are, as a rule, significantly lesser than vertical ones, so that the carbon balance (excluding the anthropogenic carbon emission) is determined by a difference between the annual uptake of CO_2 _by vegetation, and the CO_2 _emitted by the area *via *the process of decomposition of dead organic matter.

Another picture is observed in "urbanized" ecosystems (city's "green area"). A significant fraction of carbon (that in accordance with our preliminary estimation is ca 50%, which is based on O.Louks' estimation, cited in [13]), accumulated by "green area" as the annual net primary production (*NPP*), is removed from this urbanised territory and transported to either other ecosystems with different decay conditions, or through rivers to the ocean. As a result, the local balance of carbon is disturbed and the urbanised territory starts to operate as a "carbon sink". Thus, carbon is not accumulated within the territory but is instead horizontally redistributed to other areas. Generally speaking, urbanisation changes the structure of local carbon flows.

#### Total balance of carbon flows

**Figure 1:**
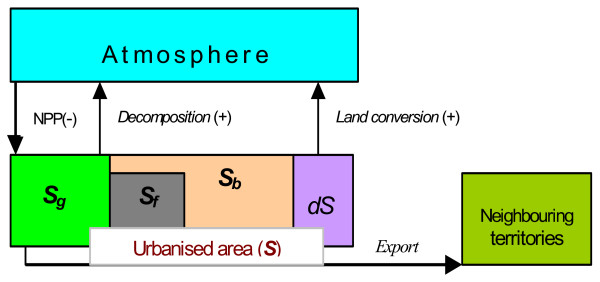
**Carbon flows in an "urbanised" ecosystem**. **Flows:**1. Production „NPP” = (*NPP*)^* ^*S*_*g*_, 2. „*Decomposition*”= =(1 - *k*_*e*_)(*NPP*)^* ^*S*_*g*_, 3. "Land conversion", *dC*_*l *_= (B^* ^+ D^*^)*dS*, **4. **"Export" = *k*_*e*_(*NPP*)^* ^*S*_*g*_. The (*NPP*)^*^, B^* ^and D^* ^are the local stationary values of the NPP, living biomass and humus, correspondingly; the coefficient *k*_*e *_is a fraction of dead organic matter exported from the "urban" ecosystem into neighbouring territories. **Areas:**1. *S*_*g *_– „Green” area (park zone), 2. *S*_*f *_– area occupied by favelas, 3. *S*_*b *_– built-up area, 4. *dS *– annual increment of urbanised area, *S *= *S*_*g *_+ *S*_*f *_+ *S*_*b *_– total urbanised area.

All the mentioned flows are shown in Fig. 1. The power of carbon sink can be estimated for all the regions. Note that our definition of sink (and source) differs from that is commonly used for the estimation of carbon balance for natural territories (see for instance [14]). The point is that in the latter, commonly used definition, sink is considered to be a system that accumulates carbon, whereas we consider sink as a system that "sucks" carbon from the environment. For instance, any through-flow system, the mass of which will not necessary increase, is, from our point of view, always a sink; whereas in accordance with the earlier definition, it is a sink only if its mass increases. It seems, that our definition is closer to the standard physical definition of sink. Let kei
 MathType@MTEF@5@5@+=feaafiart1ev1aaatCvAUfKttLearuWrP9MDH5MBPbIqV92AaeXatLxBI9gBaebbnrfifHhDYfgasaacH8akY=wiFfYdH8Gipec8Eeeu0xXdbba9frFj0=OqFfea0dXdd9vqai=hGuQ8kuc9pgc9s8qqaq=dirpe0xb9q8qiLsFr0=vr0=vr0dc8meaabaqaciaacaGaaeqabaqabeGadaaakeaacqWGRbWAdaqhaaWcbaGaemyzaugabaGaemyAaKgaaaaa@30E6@ be the share of organic carbon exported from the "urbanised" ecosystem of *j*^*th *^region into neighboring territories, and Sgj
 MathType@MTEF@5@5@+=feaafiart1ev1aaatCvAUfKttLearuWrP9MDH5MBPbIqV92AaeXatLxBI9gBaebbnrfifHhDYfgasaacH8akY=wiFfYdH8Gipec8Eeeu0xXdbba9frFj0=OqFfea0dXdd9vqai=hGuQ8kuc9pgc9s8qqaq=dirpe0xb9q8qiLsFr0=vr0=vr0dc8meaabaqaciaacaGaaeqabaqabeGadaaakeaacqWGtbWudaqhaaWcbaGaem4zaCgabaGaemOAaOgaaaaa@30BC@ = pgj
 MathType@MTEF@5@5@+=feaafiart1ev1aaatCvAUfKttLearuWrP9MDH5MBPbIqV92AaeXatLxBI9gBaebbnrfifHhDYfgasaacH8akY=wiFfYdH8Gipec8Eeeu0xXdbba9frFj0=OqFfea0dXdd9vqai=hGuQ8kuc9pgc9s8qqaq=dirpe0xb9q8qiLsFr0=vr0=vr0dc8meaabaqaciaacaGaaeqabaqabeGadaaakeaacqWGWbaCdaqhaaWcbaGaem4zaCgabaGaemOAaOgaaaaa@30F6@*S*^*j*^(*t*) be the green area of the urbanised territory. The annual balance of natural carbon between the atmosphere and urbanised territory of *j*^*th *^region will therefore be equal to:

dCsj(t)=(NPPj)*⋅Sgj(t)︸production−(1−kej)⋅(NPPj)*⋅Sgj(t)︸decomposition==kejpgjSj(t)⋅(NPPj)*,     (4)
 MathType@MTEF@5@5@+=feaafiart1ev1aaatCvAUfKttLearuWrP9MDH5MBPbIqV92AaeXatLxBI9gBaebbnrfifHhDYfgasaacH8akY=wiFfYdH8Gipec8Eeeu0xXdbba9frFj0=OqFfea0dXdd9vqai=hGuQ8kuc9pgc9s8qqaq=dirpe0xb9q8qiLsFr0=vr0=vr0dc8meaabaqaciaacaGaaeqabaqabeGadaaakeaafaqaaeGabaaabaGaemizaqMaem4qam0aa0baaSqaaiabdohaZbqaaiabdQgaQbaakiabcIcaOiabdsha0jabcMcaPiabg2da9maayaaabaGaeiikaGIaemOta4KaemiuaaLaemiuaa1aaWbaaSqabeaacqWGQbGAaaGccqGGPaqkdaahaaWcbeqaaiabcQcaQaaakiabgwSixlabdofatnaaDaaaleaacqWGNbWzaeaacqWGQbGAaaGccqGGOaakcqWG0baDcqGGPaqkaSqaaiabdchaWjabdkhaYjabd+gaVjabdsgaKjabdwha1jabdogaJjabdsha0jabdMgaPjabd+gaVjabd6gaUbGccaGL44pacqGHsisldaagaaqaaiabcIcaOiabigdaXiabgkHiTiabdUgaRnaaDaaaleaacqWGLbqzaeaacqWGQbGAaaGccqGGPaqkcqGHflY1cqGGOaakcqWGobGtcqWGqbaucqWGqbaudaahaaWcbeqaaiabdQgaQbaakiabcMcaPmaaCaaaleqabaGaeiOkaOcaaOGaeyyXICTaem4uam1aa0baaSqaaiabdEgaNbqaaiabdQgaQbaakiabcIcaOiabdsha0jabcMcaPaWcbaGaemizaqMaemyzauMaem4yamMaem4Ba8MaemyBa0MaemiCaaNaem4Ba8Maem4CamNaemyAaKMaemiDaqNaemyAaKMaem4Ba8MaemOBa4gakiaawIJ=aiabg2da9aqaaiabg2da9iabdUgaRnaaDaaaleaacqWGLbqzaeaacqWGQbGAaaGccqWGWbaCdaqhaaWcbaGaem4zaCgabaGaemOAaOgaaOGaem4uam1aaWbaaSqabeaacqWGQbGAaaGccqGGOaakcqWG0baDcqGGPaqkcqGHflY1cqGGOaakcqWGobGtcqWGqbaucqWGqbaudaahaaWcbeqaaiabdQgaQbaakiabcMcaPmaaCaaaleqabaGaeiOkaOcaaOGaeiilaWcaaiaaxMaacaWLjaWaaeWaceaacqaI0aanaiaawIcacaGLPaaaaaa@A720@

where (*NPP*^*j*^)^* ^is some mean value of the annual NPP of the corresponding regional natural ecosystems, *S*^*j*^(*t*) is the area of urbanised territory and pgj
 MathType@MTEF@5@5@+=feaafiart1ev1aaatCvAUfKttLearuWrP9MDH5MBPbIqV92AaeXatLxBI9gBaebbnrfifHhDYfgasaacH8akY=wiFfYdH8Gipec8Eeeu0xXdbba9frFj0=OqFfea0dXdd9vqai=hGuQ8kuc9pgc9s8qqaq=dirpe0xb9q8qiLsFr0=vr0=vr0dc8meaabaqaciaacaGaaeqabaqabeGadaaakeaacqWGWbaCdaqhaaWcbaGaem4zaCgabaGaemOAaOgaaaaa@30F6@ = 0.3(1 - *f*^*j*^) is its green fraction. Although, we have estimated kei
 MathType@MTEF@5@5@+=feaafiart1ev1aaatCvAUfKttLearuWrP9MDH5MBPbIqV92AaeXatLxBI9gBaebbnrfifHhDYfgasaacH8akY=wiFfYdH8Gipec8Eeeu0xXdbba9frFj0=OqFfea0dXdd9vqai=hGuQ8kuc9pgc9s8qqaq=dirpe0xb9q8qiLsFr0=vr0=vr0dc8meaabaqaciaacaGaaeqabaqabeGadaaakeaacqWGRbWAdaqhaaWcbaGaemyzaugabaGaemyAaKgaaaaa@30E6@ to be equal to 0.5, this value may vary for different regions. As it may be difficult to explicitly estimate the regional values, we assume that kei
 MathType@MTEF@5@5@+=feaafiart1ev1aaatCvAUfKttLearuWrP9MDH5MBPbIqV92AaeXatLxBI9gBaebbnrfifHhDYfgasaacH8akY=wiFfYdH8Gipec8Eeeu0xXdbba9frFj0=OqFfea0dXdd9vqai=hGuQ8kuc9pgc9s8qqaq=dirpe0xb9q8qiLsFr0=vr0=vr0dc8meaabaqaciaacaGaaeqabaqabeGadaaakeaacqWGRbWAdaqhaaWcbaGaemyzaugabaGaemyAaKgaaaaa@30E6@ = 0.5 for all regions.

However, in reality urbanised territories are expanding, hence *dS*^*j *^(*t*) ≠ 0. This term, expressing the dynamics of urbanised territories, should be taken into account within the general expression for the total annual balance of carbon, dCtotj
 MathType@MTEF@5@5@+=feaafiart1ev1aaatCvAUfKttLearuWrP9MDH5MBPbIqV92AaeXatLxBI9gBaebbnrfifHhDYfgasaacH8akY=wiFfYdH8Gipec8Eeeu0xXdbba9frFj0=OqFfea0dXdd9vqai=hGuQ8kuc9pgc9s8qqaq=dirpe0xb9q8qiLsFr0=vr0=vr0dc8meaabaqaciaacaGaaeqabaqabeGadaaakeaacqWGKbazcqWGdbWqdaqhaaWcbaGaemiDaqNaem4Ba8MaemiDaqhabaGaemOAaOgaaaaa@34DF@:

dCtotj(t)=dClj(t)−dCsj(t)=(0.7+0.3fj)dSi(t)⋅[(Bj)*+(Dj)*]−−0.3kej(1−fj)Sj(t)⋅(NPPj)*.     (5)
 MathType@MTEF@5@5@+=feaafiart1ev1aaatCvAUfKttLearuWrP9MDH5MBPbIqV92AaeXatLxBI9gBaebbnrfifHhDYfgasaacH8akY=wiFfYdH8Gipec8Eeeu0xXdbba9frFj0=OqFfea0dXdd9vqai=hGuQ8kuc9pgc9s8qqaq=dirpe0xb9q8qiLsFr0=vr0=vr0dc8meaabaqaciaacaGaaeqabaqabeGadaaakeaafaqabeGabaaabaGaemizaqMaem4qam0aa0baaSqaaiabdsha0jabd+gaVjabdsha0bqaaiabdQgaQbaakiabcIcaOiabdsha0jabcMcaPiabg2da9iabdsgaKjabdoeadnaaDaaaleaacqWGSbaBaeaacqWGQbGAaaGccqGGOaakcqWG0baDcqGGPaqkcqGHsislcqWGKbazcqWGdbWqdaqhaaWcbaGaem4CamhabaGaemOAaOgaaOGaeiikaGIaemiDaqNaeiykaKIaeyypa0JaeiikaGIaeGimaaJaeiOla4IaeG4naCJaey4kaSIaeGimaaJaeiOla4IaeG4mamJaemOzay2aaWbaaSqabeaacqWGQbGAaaGccqGGPaqkcqWGKbazcqWGtbWudaahaaWcbeqaaiabdMgaPbaakiabcIcaOiabdsha0jabcMcaPiabgwSixpaadmGabaGaeiikaGIaemOqai0aaWbaaSqabeaacqWGQbGAaaGccqGGPaqkdaahaaWcbeqaaiabcQcaQaaakiabgUcaRiabcIcaOiabdseaenaaCaaaleqabaGaemOAaOgaaOGaeiykaKYaaWbaaSqabeaacqGGQaGkaaaakiaawUfacaGLDbaacqGHsislaeGaceqWdeq8diabgkHiTiabicdaWiabc6caUiabiodaZiabdUgaRnaaDaaaleaacqWGLbqzaeaacqWGQbGAaaGccqGGOaakcqaIXaqmcqGHsislcqWGMbGzdaahaaWcbeqaaiabdQgaQbaakiabcMcaPiabdofatnaaCaaaleqabaGaemOAaOgaaOGaeiikaGIaemiDaqNaeiykaKIaeyyXICTaeiikaGIaemOta4KaemiuaaLaemiuaa1aaWbaaSqabeaacqWGQbGAaaGccqGGPaqkdaahaaWcbeqaaiabcQcaQaaakiabc6caUaaacaWLjaGaaCzcamaabmGabaGaeGynaudacaGLOaGaayzkaaaaaa@940D@

This formula represents the local carbon balance, namely, the one for a given territory. It is obvious that, if dCtotj
 MathType@MTEF@5@5@+=feaafiart1ev1aaatCvAUfKttLearuWrP9MDH5MBPbIqV92AaeXatLxBI9gBaebbnrfifHhDYfgasaacH8akY=wiFfYdH8Gipec8Eeeu0xXdbba9frFj0=OqFfea0dXdd9vqai=hGuQ8kuc9pgc9s8qqaq=dirpe0xb9q8qiLsFr0=vr0=vr0dc8meaabaqaciaacaGaaeqabaqabeGadaaakeaacqWGKbazcqWGdbWqdaqhaaWcbaGaemiDaqNaem4Ba8MaemiDaqhabaGaemOAaOgaaaaa@34DF@ > 0, then the given territory is a source of carbon, while if dCtotj
 MathType@MTEF@5@5@+=feaafiart1ev1aaatCvAUfKttLearuWrP9MDH5MBPbIqV92AaeXatLxBI9gBaebbnrfifHhDYfgasaacH8akY=wiFfYdH8Gipec8Eeeu0xXdbba9frFj0=OqFfea0dXdd9vqai=hGuQ8kuc9pgc9s8qqaq=dirpe0xb9q8qiLsFr0=vr0=vr0dc8meaabaqaciaacaGaaeqabaqabeGadaaakeaacqWGKbazcqWGdbWqdaqhaaWcbaGaemiDaqNaem4Ba8MaemiDaqhabaGaemOAaOgaaaaa@34DF@ < 0, then it is a sink. If dCtotj
 MathType@MTEF@5@5@+=feaafiart1ev1aaatCvAUfKttLearuWrP9MDH5MBPbIqV92AaeXatLxBI9gBaebbnrfifHhDYfgasaacH8akY=wiFfYdH8Gipec8Eeeu0xXdbba9frFj0=OqFfea0dXdd9vqai=hGuQ8kuc9pgc9s8qqaq=dirpe0xb9q8qiLsFr0=vr0=vr0dc8meaabaqaciaacaGaaeqabaqabeGadaaakeaacqWGKbazcqWGdbWqdaqhaaWcbaGaemiDaqNaem4Ba8MaemiDaqhabaGaemOAaOgaaaaa@34DF@ = 0 then the territory is neutral with respect to the GCC.

**Figure 2:**
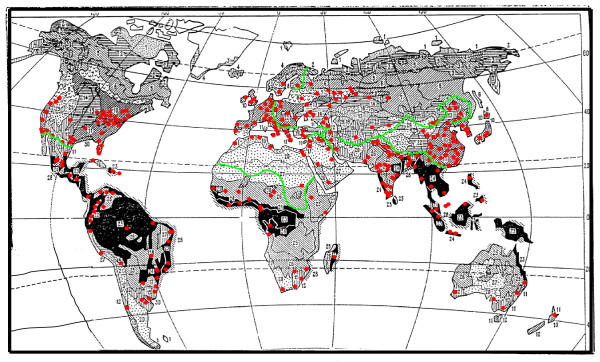
**Bazilevich's biomes map**. The biomes numbering is shown in Tab. 3. Domains with urbanised population are marked by red points.

If we look at Eq. (5), we can see that the total carbon flow depends, in general, on two groups of parameters. The first includes the NPP, (*NPP*^*j*^)^*^, and the sum of living biomass and dead organic matter, (*B*^*j*^)^* ^+ (*D*^*J*^)^*^, expressed in carbon units. We estimated them by two different methods based on two concepts of the spatial distribution of local urbanised territories over a region. One of them, stating that the cities are randomly distributed over the territory of a region, was used earlier in [1]. Another concept is more realistic, since it takes into account the fact the distribution of urbanised territories over regions is not homogeneous or random. It is visibly seen in Fig. 2, in which red-marked cities are represented on the global biome map. It is obviously the cities are historically attracted to domains that are more suitable for human life conditions (local climate, vegetation, soil, etc). In a certain sense, all these factors are reflected by integral parameters such as the productivity of the local vegetation, its living biomass, and the storage of dead organic matter. The second group of parameters deals with the areas of urban territories, *S*^*j*^(*t*), and their annual increments, *dS*^*i*^(*t*). The problem of how to estimate these latter parameters will be considered in the future article.

#### Estimation of mean regional values of the NPP, living biomass, and dead organic matter

Results of estimations are contained in Tabs. 3 and 4.: Tab. 3 contains the data corresponding to the assumption about uniform spatial distribution of cities over the territory of a whole region; while the data corresponding to a localized city's spatial distribution over the regional territory is in Tab. 4. (see Fig. 2).

**Table 3:** Annual regional means for the net primary production (NPP^*^, in 10^3^**tonsC/km**^2^per year), and the sum of specific living biomass and dead organic matter – humus (B^* ^+ D^*^, in 10^3^**tonsC/km**^2^): random model.

*Region*	*Afr*	*Ar*	*AsP*	*Cn*	*LAC*	*ET*	*HI*	*UCA*
NPP^*^	0.80	0.24	0.82	0.34	0.72	0.31	0.50	0.53
B^* ^+ D^*^	21.8	7.4	24.5	19.4	23.3	13.2	23.8	22.9

**Table 4:** Means of the NPP (in 10^3 ^tons C/km^2^per year), and the sum of specific living biomass and dead organic matter (in 10^3 ^tons C/km^2^) for urbanised territories

***Region***	***Afr***	***Ar***	***AsP***	***Cn***	***LAC***	***ET***	***HI***	***UCA***
NPPu* MathType@MTEF@5@5@+=feaafiart1ev1aaatCvAUfKttLearuWrP9MDH5MBPbIqV92AaeXatLxBI9gBaebbnrfifHhDYfgasaacH8akY=wiFfYdH8Gipec8Eeeu0xXdbba9frFj0=OqFfea0dXdd9vqai=hGuQ8kuc9pgc9s8qqaq=dirpe0xb9q8qiLsFr0=vr0=vr0dc8meaabaqaciaacaGaaeqabaqabeGadaaakeaacqWGobGtcqWGqbaucqWGqbaudaqhaaWcbaGaemyDauhabaGaeiOkaOcaaaaa@329F@	0.70	0.25	0.72	0.56	0.66	0.33	0.50	0.56
Bu*+Du* MathType@MTEF@5@5@+=feaafiart1ev1aaatCvAUfKttLearuWrP9MDH5MBPbIqV92AaeXatLxBI9gBaebbnrfifHhDYfgasaacH8akY=wiFfYdH8Gipec8Eeeu0xXdbba9frFj0=OqFfea0dXdd9vqai=hGuQ8kuc9pgc9s8qqaq=dirpe0xb9q8qiLsFr0=vr0=vr0dc8meaabaqaciaacaGaaeqabaqabeGadaaakeaacqWGcbGqdaqhaaWcbaGaemyDauhabaGaeiOkaOcaaOGaey4kaSIaemiraq0aa0baaSqaaiabdwha1bqaaiabcQcaQaaaaaa@34AE@	18.2	8.4	23.3	27.9	20.6	21.0	28.7	24.0

## Discussion

If we compare the data from these tables, we can see that for almost all the regions (except Cn and ET) the values of NPP^* ^and B^* ^+ D^*^, that were estimated using both models, do not significantly differ from each other, although in such "tropical" regions as Afr, AsP and LAC, there is a tendency for attraction towards more "moderate" locations that is manifested in a decrease of the NPP values. But if we neglect this shift, we can say that in all these regions, cities are distributed over their territories almost randomly.

As for the Cn region, then the significant deviation from the random model can be explained by the fact that 4/5 of China's territory is unpopulated semi- and full desert, hence it is natural that Chinese cities are "attracted" to more productive territories. The ET region is characterised by a significant shift of the living biomass and humus storage in the direction of their greater values. If we take into account that the ET region represented mainly by the territory of the former USSR, then the historical explanation could become relevant here. [15]. Historically, because of the large distances and poorly developed transport network, each Russian city needed its own food supply, hence would be surrounded by a "ring" of agricultural lands. Productive agriculture, in turn, requires fertile soil. The latter is characterised by a high value of humus, maintained by the abundance of living biomass (for example, the famous Chernozem belt of Russia).

## Conclusion

As a result, we have built the base model for the cities' structure in relation to its role in the local carbon cycle and also calculated the regional weighted means of productivity, living biomass and dead organic matter for urbanised territories. In the framework of current climate policies, it becomes more and more important to be able to forecast these parameters, as well as the dynamics of regional urban areas. Although one method of dynamic forecasting of these parameters, based on the statistical regression model, was already suggested [1], nevertheless we shall further develop a new technique based on Sir R. Fisher's idea to use the gamma-distribution. This will allow us to calculate the total carbon balance, show how urbanization shifts it and see whether the regions are going to act as sources or sinks of anthropogenic carbon in the course of the next 100 years.

## Methods

In order to estimate the values of NPP^* ^and (B^* ^+D^*^), we use Bazilevich's global data set [16], applying the smoothing and correction procedure [17]. The elementary unit of the database is a biome. A list of all main biomes is presented in Tab. 5.

**Table 5:** Different types of global vegetation (biomes)

*1. Polar desert, polar tundra*	*16. Dry steppe*
*2. Tundra*	*17. Sub-boreal desert*
*3. Mountainous tundra*	*18. Sub-boreal saline desert*
*4. Forest tundra*	*19. Subtropical semi-desert*
*5. North taiga*	*20. Subtropical desert*
*6. Middle taiga*	*21. Mountainous desert*
*7. South taiga*	*22. Alpine and Sub-alpine meadows*
*8. Temperate mixed forest*	*23. Evergreen tropical rain forest*
*9. Aspen-Birch lower taiga*	*24. Deciduous tropical forest*
*10. Deciduous forest*	*25. Tropical xerophyte woodland*
*11. Subtropical deciduous and coniferous forest*	*26. Tropical savannah*
*12. Xerophyte woods and shrubs*	*27. Tropical desert*
*13. Forest steppe*	*28. Mangrove forest*
*14. Temperate dry steppe (including mountainous)*	*29. Saline land*
*15. Savannah*	*30. Subtropical and tropical woodland, tugaj shrubs*

In addition, the data for *NPP *and the densities of living biomass and dead organic matter (humus) for the main biomes is presented in Tab. 6. A geographical explication of these biomes (Bazilevich's biomes map) is shown in Fig. 2. The regional borders and the domains with urbanised population (marked by red points) are also presented in this figure. It is obvious that the borders of biomes do not coincide with the borders of states, UN regions or urbanised territories.

**Table 6:** Annual net primary production, NPP^* ^(10^3 ^tons C/km^2^·year, density of living biomass, B^* ^(10^3 ^tons C/km^2^, and density of dead organic matter, D^* ^(10^3^tons C/(km^2^, in 1 m soil); *a *- biome type, *b *- biome area (× 10^6^km^2^). Biomes n°9 and n°28 are not included because of the smallness of their territories. *Source: *Svirezhev (2002).

***a***	***B***	NPP^*^	B^*^	D^*^	***a***	***b***	NPP^*^	B^*^	D^*^
**1**	2.55	0.068	0.148	0.938	**16**	2.66	0.15	0.32	7.04
**2**	2.93	0.144	0.76	3.08	**17**	2.08	0.18	0.45	6.8
**3**	2.23	0.15	0.76	3.06	**18**	2.59	0.096	0.18	4.56
**4**	1.55	0.26	1.5	5.02	**19**	1.99	0.14	0.32	4.94
**5**	5.45	0.22	3.2	4.52	**20**	7.16	0.044	0.096	0.87
**6**	5.73	0.25	6.2	6.06	**21**	1.15	0.18	0.32	9.49
**7**	6.60	0.26	7.4	11.5	**22**	3.54	0.3	0.76	13.4
**8**	2.12	0.35	8.0	16.1	**23**	10.4	1.3	18.0	13.4
**10**	7.21	0.53	15.0	16.9	**24**	7.81	0.95	16.0	13.1
**11**	5.75	0.71	14.2	14.4	**25**	9.18	0.54	2.4	10.6
**12**	3.91	0.23	1.5	8.4	**26**	17.1	0.5	2.4	10.2
**13**	3.72	0.3	0.76	23.3	**27**	11.5	0.068	0.144	1.4
**14**	4.29	0.32	0.76	18.1	**29**	0.37	0.068	0.15	2.75
**15**	1.66	0.44	1.5	14.8	**30**	0.9	0.78	16.0	12.1

If we superimpose the sufficiently fine grid (so that each cell contains not more than a single red point) on the biomes map, then we can construct so-called "biome portrait" of a regional urban territory. For this we have to calculate the percentage of urban area that is occupied by every biome, (πu)kj(∑130(πu)kj=1)
 MathType@MTEF@5@5@+=feaafiart1ev1aaatCvAUfKttLearuWrP9MDH5MBPbIqV92AaeXatLxBI9gBaebbnrfifHhDYfgasaacH8akY=wiFfYdH8Gipec8Eeeu0xXdbba9frFj0=OqFfea0dXdd9vqai=hGuQ8kuc9pgc9s8qqaq=dirpe0xb9q8qiLsFr0=vr0=vr0dc8meaabaqaciaacaGaaeqabaqabeGadaaakeaacqGGOaakcqaHapaCdaWgaaWcbaGaemyDauhabeaakiabcMcaPmaaDaaaleaacqWGRbWAaeaacqWGQbGAaaGcdaqadiqaamaaqadabaGaeiikaGIaeqiWda3aaSbaaSqaaiabdwha1bqabaGccqGGPaqkdaqhaaWcbaGaem4AaSgabaGaemOAaOgaaOGaeyypa0JaeGymaedaleaacqaIXaqmaeaacqaIZaWmcqaIWaama0GaeyyeIuoaaOGaayjkaiaawMcaaaaa@4521@. These portraits for each of the eight regions are represented in Tab. 7. Note, that the number of cells covering each region is adequate and rather high: for instance, the Afr region is covered by 155 cells, the UCA by 340 cells, and even a relatively small region such as the HI contains 62 cells.

**Table 7:** The biome portraits of urbanised territories for different regions. Fractions (πu)kj
 MathType@MTEF@5@5@+=feaafiart1ev1aaatCvAUfKttLearuWrP9MDH5MBPbIqV92AaeXatLxBI9gBaebbnrfifHhDYfgasaacH8akY=wiFfYdH8Gipec8Eeeu0xXdbba9frFj0=OqFfea0dXdd9vqai=hGuQ8kuc9pgc9s8qqaq=dirpe0xb9q8qiLsFr0=vr0=vr0dc8meaabaqaciaacaGaaeqabaqabeGadaaakeaacqGGOaakcqaHapaCdaWgaaWcbaGaemyDauhabeaakiabcMcaPmaaDaaaleaacqWGRbWAaeaacqWGQbGAaaaaaa@34AD@ is expressed in %.

***Region\Biome***	***Afr***	***Ar***	***AsP***	***Cn***	***LAC***	***ET***	***HI***	***UCA***
*6. Middle taiga*						4.0	3.1	
*7. South taiga*						23.3	3.0	
*8. Temperate mixed forest*						11.6	5.8	2.2
*10. Deciduous forest*			8.5	42.9		19.6	67.6	13.2
*9. Subtropical deciduous & coniferous forest*		15.3	17.0	42.9	15.6		11.8	62.9
*12. Xeropyte woods & shrubs*	3.8		2.1					6.1
*13. Forest steppe*				4.8		11.7		2.1
*14. Temperate dry steppe*			2.1		6.3	19.0	2.8	1.8
*15. Savannah*	11.5				9.4			
*16. Dry steppe*						3.8		
*17. Subboreal desert*					6.2			5.9
*18. Subboreal saline desert*						2.7		
*19. Subtropical semi-desert*		30.8	2.2					
*20. Subtropical desert*		30.8						1.9
*22. Alpine & subalpine meadows*				4.8	12.5	3.9	5.9	
*23. Evergreen tropic rain forest*	26.9		10.6		21.9			
*24. Deciduous tropical forest*	3.8		14.9		6.3			3.9
*25. Tropical xerophyte woodland*	11.5		23.0					
*26. Tropical savannah*	38.5	15.4	6.4		12.5			
*27. Tropical desert*	3.8	7.7			6.2			
*29. Saline land*				4.8	3.1			
*30. Tropical & subtropical woodland & tugaj*			12.8					

Having constructed the regional biome portraits of urbanised territories, we can next calculate the regional weighting means of productivity, living biomass and dead organic matter for each *j*^*th *^region as

(NPPu*)j=∑k=130(πu)kj(NPP*)k, (Bu*)j=∑k=130(πu)kj(B*)k, (Du*)j=∑k=130(πu)kj(D*)k,     (6)
 MathType@MTEF@5@5@+=feaafiart1ev1aaatCvAUfKttLearuWrP9MDH5MBPbIqV92AaeXatLxBI9gBaebbnrfifHhDYfgasaacH8akY=wiFfYdH8Gipec8Eeeu0xXdbba9frFj0=OqFfea0dXdd9vqai=hGuQ8kuc9pgc9s8qqaq=dirpe0xb9q8qiLsFr0=vr0=vr0dc8meaabaqaciaacaGaaeqabaqabeGadaaakeaacqGGOaakcqWGobGtcqWGqbaucqWGqbaudaqhaaWcbaGaemyDauhabaGaeiOkaOcaaOGaeiykaKYaaWbaaSqabeaacqWGQbGAaaGccqGH9aqpdaaeWbqaaiabcIcaOiabec8aWnaaBaaaleaacqWG1bqDaeqaaOGaeiykaKYaa0baaSqaaiabdUgaRbqaaiabdQgaQbaaaeaacqWGRbWAcqGH9aqpcqaIXaqmaeaacqaIZaWmcqaIWaama0GaeyyeIuoakiabcIcaOiabd6eaojabdcfaqjabdcfaqjabcQcaQiabcMcaPmaaBaaaleaacqWGRbWAaeqaaOGaeiilaWIaeeiiaaIaeiikaGIaemOqai0aa0baaSqaaiabdwha1bqaaiabcQcaQaaakiabcMcaPmaaCaaaleqabaGaemOAaOgaaOGaeyypa0ZaaabCaeaacqGGOaakcqaHapaCdaWgaaWcbaGaemyDauhabeaakiabcMcaPmaaDaaaleaacqWGRbWAaeaacqWGQbGAaaaabaGaem4AaSMaeyypa0JaeGymaedabaGaeG4mamJaeGimaadaniabggHiLdGccqGGOaakcqWGcbGqcqGGQaGkcqGGPaqkdaWgaaWcbaGaem4AaSgabeaakiabcYcaSiabbccaGiabcIcaOiabdseaenaaDaaaleaacqWG1bqDaeaacqGGQaGkaaGccqGGPaqkdaahaaWcbeqaaiabdQgaQbaakiabg2da9maaqahabaGaeiikaGIaeqiWda3aaSbaaSqaaiabdwha1bqabaGccqGGPaqkdaqhaaWcbaGaem4AaSgabaGaemOAaOgaaaqaaiabdUgaRjabg2da9iabigdaXaqaaiabiodaZiabicdaWaqdcqGHris5aOGaeiikaGIaemiraqKaeiOkaOIaeiykaKYaaSbaaSqaaiabdUgaRbqabaGccqGGSaalcaWLjaGaaCzcamaabmGabaGaeGOnaydacaGLOaGaayzkaaaaaa@8F17@

where the values of NPP^*^, B^* ^and D^* ^are taken from Tab. 6.

If we employ the concept of uniform, homogeneous spatial distribution of cities over the whole territory of a given region, then we have to construct the regional biome portrait, as in [1]. However, here we assume that in the process of urbanisation, humans prefer to master (with certain regional coefficients of preference) only those domains that are similar (in respect to a biome's portrait) to the domains that have already been mastered in the past.

## Competing interests

The author(s) declare that they have no competing interests.

## Authors' contributions

ASH conceived the concept, carried out the studies, wrote the manuscript.

HJS participated in coordination, conceptual analysis and editing of the manuscript.

All authors read and approved the final manuscript.
